# Evaluation of the binding interactions between *Plasmodium falciparum* Kelch-13 mutant recombinant proteins with artemisinin

**DOI:** 10.1371/journal.pone.0306975

**Published:** 2024-08-15

**Authors:** Noorazian Md. Yusuf, Aisya Nazura Azman, Amirul Adli Abdul Aziz, Fazia Adyani Ahmad Fuad, Ruhayatun Naimah Nasarudin, Shamilah Hisam

**Affiliations:** 1 Parasitology Unit, Infectious Disease Research Centre, Institute for Medical Research, National Institute of Health, Shah Alam, Malaysia; 2 Department of Chemical Engineering & Sustainability, Faculty of Engineering, International Islamic University Malaysia, Kuala Lumpur, Malaysia; 3 School of Biology, Faculty of Applied Sciences, Universiti Teknologi MARA (UiTM) Cawangan Negeri Sembilan, Kampus Kuala Pilah, Kuala Pilah, Malaysia; University of Cape Coast, GHANA

## Abstract

Malaria, an ancient mosquito-borne illness caused by *Plasmodium* parasites, is mostly treated with Artemisinin Combination Therapy (ACT). However, Single Nucleotide Polymorphisms (SNPs) mutations in the *P*. *falciparum* Kelch 13 (*PfK13*) protein have been associated with artemisinin resistance (ART-R). Therefore, this study aims to generate *PfK13* recombinant proteins incorporating of two specific SNPs mutations, *PfK13*-V494I and *PfK13*-N537I, and subsequently analyze their binding interactions with artemisinin (ART). The recombinant proteins of *PfK13* mutations and the Wild Type (WT) variant were expressed utilizing a standard protein expression protocol with modifications and subsequently purified via IMAC and confirmed with SDS-PAGE analysis and Orbitrap tandem mass spectrometry. The binding interactions between *PfK13*-V494I and *PfK13*-N537I propeller domain proteins ART were assessed through Isothermal Titration Calorimetry (ITC) and subsequently validated using fluorescence spectrometry. The protein concentrations obtained were 0.3 mg/ml for *PfK13*-WT, 0.18 mg/ml for *PfK13*-V494I, and 0.28 mg/ml for *PfK13*-N537I. Results obtained for binding interaction revealed an increased fluorescence intensity in the mutants *PfK13*-N537I (83 a.u.) and *PfK13*-V494I (143 a.u.) compared to *PfK13*-WT (33 a.u.), indicating increased exposure of surface proteins because of the looser binding between *PfK13* protein mutants with ART. This shows that the *PfK13* mutations may induce alterations in the binding interaction with ART, potentially leading to reduced effectiveness of ART and ultimately contributing to ART-R. However, this study only elucidated one facet of the contributing factors that could serve as potential indicators for ART-R and further investigation should be pursued in the future to comprehensively explore this complex mechanism of ART-R.

## Introduction

*Plasmodium* parasites are the cause of malaria, a disease that continues to be one of the biggest threats to global health, especially in tropical and subtropical areas. Malaria places a significant burden on public health systems around the world, with 249 million cases recorded in 2022. Between 2000 and 2019, the predicted mortality rate decreased from 28.8%. It continued to reduce, reaching 15.2 in 2015 and 14.3 in 2022. But given that children under the age of five account for most cases, this cannot be dismissed, thus malaria remained as a major global burden on public health systems [1]. *Plasmodium falciparum* is the deadliest species of the malaria parasite, responsible for most malaria-related deaths. Its ability to develop resistance to antimalarial drugs, including chloroquine [2–5] and sulfadoxine-pyrimethamine [6–8], has further complicated treatment efforts. Artemisinin-based combination therapies (ACTs) were introduced by the World Health Organisation (WHO) as the frontline treatment for uncomplicated falciparum malaria, offering rapid parasite clearance and reducing the risk of resistance development [9]. ACTs are the combination of a fast-acting artemisinin derivative with a more slowly eliminated anti-malarial drug of a different class. The role of the artemisinin component is to act rapidly and reduce the number of parasites during the first 3-days of treatment, while the role of partner drugs is to eliminate the remaining parasite while protecting the development of parasite resistance against artemisinin [10]. Artemisinin, derived from the sweet wormwood plant (Artemisia annua), exhibits potent antimalarial properties, making it a cornerstone in malaria treatment strategies [11].

The deployment of ACT in Malaysia for uncomplicated malaria started in 2013, following the “Management Guidelines of Malaria in Malaysia, 2014 [12].

The effectiveness of antimalarial medications is assessed via therapeutic efficacy studies (TES) or DRS (Drug Resistance Surveillance- for Malaysia), which observe clinical and parasitological outcomes in individuals undergoing antimalarial treatment. TES/DRS serves as the benchmark for countries to establish their national treatment policies most effectively. Various methods can be employed to evaluate resistance to antimalarial drugs. In the case of certain medications, genetic alterations linked to diminished sensitivity have been pinpointed. The tracking of partial resistance to artemisinin involves using a recognized set of validated and potential *Pf*Kelch13 markers, which are linked to delayed clearance following artemisinin-based treatments [10].

However, despite the initial effectiveness of artemisinin, the emergence and spread of artemisinin-resistant *Plasmodium* strains, particularly in Southeast Asia, pose a significant threat to malaria control and elimination efforts globally. The mechanisms underlying artemisinin resistance are complex and multifaceted, involving genetic mutations in various parasite proteins, including the Kelch-13 (*PfK13*) propeller domain. The first cases of artemisinin resistance (ART-R) were detected in Cambodia in 2008 [13, 14] and spread throughout Southeast Asia and the African region, including Myanmar [15, 16], Thailand [17], Cambodia [18], Papua New Guinea (PNG) [19], Vietnam [20], Southern China [21], India [22], African countries [23]. Menard and his colleagues (MalariaGEN *Plasmodium falciparum* Community Project) [24, 25] have done tremendous work mapping the *K13*-propeller polymorphism in 59 countries where malaria was endemic. They identified 108 non-synonymous *K13* mutations with a specific distribution of ART-R.

Witkowski and colleagues [26] conducted a comparative analysis of the exome of an African strain (F32-Tanzania) and a drug-resistant strain (F32-ART), which had been repeatedly exposed to increasing drug concentrations over a period of five years. They compared the exome of F32-ART with its sibling clone (F32-TEM), cultured under identical conditions but without drug exposure. Through analysis of single nucleotide polymorphisms (SNPs) in the exomes, they identified eight non-synonymous SNPs in seven genes. Further investigation involved analyzing the genomes of intermediate strains (after 22, 40, or 56 cycles of exposure) and 49 isolates from Cambodia. This analysis uncovered a significant correlation between mutations in the propeller domain of the Kelch gene, located on chromosome 13 (*K13*, *PF*3D7_1343700).

In Malaysian settings, *PfK13* was identified via molecular surveillance of patient samples in Malaysian Borneo (Sabah) spanning from 2012 to 2016, and in Peninsular Malaysia from 2008 to 2017 [27]. While numerous mutations in the *PfK13* propeller region have been identified, there is no suspected or confirmed evidence of endemic artemisinin-resistant *P*. *falciparum* in this pre-elimination context in Malaysia. Therefore, the current guidelines advocating for first-line treatment with artemisinin-based combination therapy (ACT) remain suitable for managing uncomplicated malaria in Malaysia.

Kelch 13 (*K13*) protein is a 726 amino acid protein that has been identified as a marker for artemisinin resistance in *P*. *falciparum* [28, 29]. According to WHO guidelines, artemisinin resistance is described as delayed parasite clearance after treatment with artesunate alone or in combination with artemisinin-based therapies [30]. WHO listed several Single Nucleotide Polymorphisms (SNPs) which were validated and confirmed to be associated with ART-R [30].

ART and its derivatives are activated by decreased heme produced by hemoglobin digestion [31–33]. ART induces extensive protein degradation and impairs proteasome function, resulting in proteotoxic stress and, eventually, parasite death [34–36]. During ART treatment, wild-type (WT) *PfK13* binds to *Pf*PI3K, resulting in *Pf*PI3K ubiquitination and degradation, eventually leading to parasite death. In contrast, mutant *PfK13* does not bind to *Pf*PI3K, activating PI3P signaling and supporting parasite life [37]. Even though the function of the *K13* protein is unknown, computational studies on its mechanism indicate that it may be involved in the unfolded protein response (UPR) signaling pathway, protein folding, protein binding, translation, and oxidative stress responses [38]. Numerous theories have been proposed to elucidate the mechanism of ART-R in parasites. For instance, the investigation conducted by Fairhurst and colleagues delineates two theories regarding the mechanisms that contribute to ART resistance. In the initial scenario involving ART-sensitive parasites, the *K13* protein binds to the transcription factor [39]. However, in cases of resistance, the parasite detaches from the transcription factor, resulting in an escalation of parasite proliferation. In the second scenario observed in ART-sensitive parasites, the *K13* protein binds to the P13K protein. Upon the development of resistance, it dissociates from P13K, leading to an increase in P13P and consequently placing the parasite in a protected state that enables survival during ART treatment [37]. Previous research has also noted that ART functions by elevating the parasite’s oxidative stress, resulting in the impairment of proteins within the unfolded protein response (UPR) pathway [40]. However, in cases where the parasite develops resistance to ART, the generation of damaged proteins decreases, enabling the parasite to withstand ART treatment. This hypothesis finds support in another study suggesting that ART exerts a dual effect by inducing protein damage and inhibiting the proteasome, thereby promoting the buildup of ubiquitinated proteins. Nonetheless, resistant parasites evade this mechanism, ultimately ensuring their survival during ART therapy [38].

However, a fascinating investigation conducted in 2020 by Birnbaum and associates suggests that the endocytosis pathway outlined by K13 is required for the uptake of hemoglobin from host cells [41]. Additionally, they demonstrate how endocytosis is connected to the K13 compartment (cellular structure) and the KICs (Kelch 13 interaction Candidates) proteins that interact with it. It is possible that the KICs regulate the degree of endocytosis, which in turn affects the quantity of hemoglobin that can be broken down and, ultimately, the amount of the active medication. Eight KIC proteins, including K13, can be rendered inactive to reduce parasite resistance. Yet, analysis of the mutant K13 C580Y showed that its decreased overall activity is the most likely mechanism of how K13 mutation causes resistance rather than altering any aspect of its function, indicating that decreased K13 activity leads to ART-R resistance.

It is known that ART medications cause *P*. *falciparum* to experience oxidative stress and cellular damage; however, it is still unclear how the mutant *PfK13* protein enables the parasites to withstand this oxidative attack. *PfK13* is necessary for the survival of parasites. Thus, it is essential to comprehend the molecular interactions between artemisinin and its target proteins, including *PfK13*, to unravel the processes of resistance and create new therapeutic approaches to counteract resistant strains of malaria. Using a recombinant version of specific *PfK13* protein mutants, this manuscript attempts to assess the binding interactions between recombinant *PfK13* mutant proteins and artemisinin to provide some understanding of the molecular basis of artemisinin resistance and to guide the development of future antimalarial therapies.

## Materials and methods

### Generations of the *PfK13*-N537I and *PfK13*-V494I recombinant constructs

Two mutation sequences N537I and V494I were selected from a previous study [27] with the criteria of: (i) The mutation was observed in two of our samples, unlike others.; (ii) At the time of our report [27] this particular sample was exclusive to our dataset, rendering it unique to our study and deserving further investigation and (iii) As per our previous in-silico study, V494I emerged as a potential candidate for artemisinin resistance (ART-R) [38]. The *PfK13* gene with the insertion of two mutations generated via Site-directed Mutagenesis (SDM) namely as *PfK13*-N537I and *PfK13*-V494I were obtained from Noor Azian MY (National Institute of Health, NIH, Malaysia: Unpublished data). The *PfK13*-Wild type (*PfK13*-WT) was used as a control. These sequences underwent amplification via a one-step PCR in 50μl volume consisting of 200ng DNA, 20 μM each primer (*K13*F-2BT: 5’ TACTTCCAATCCAATGCACCTTTTCCTTTGGTTTTC 3’ / *K13*R-2BT: 5’ TTATCCACTTCCAATGTTATTATATGTTAGCAATTAGTACTGAATGTCC 3’) and MyTaq Mix 2x (Bioline) with cycling conditions of initial denaturation at 95oC, 34 cycles of 95oC, 51oC and 72oC with final extension at 72oC, followed by ligation into the pET His6-TEV-LIC cloning vector (2B-T) at BamHI sites and subsequent transformation into NovaBlue GigaSingle™ competent cells (Novagen). Following plating on an agar plate with 100 μg/ml of Ampicillin, both mutant and WT constructs were incubated overnight at 37°C. Sequencing reactions were carried out by (Apical Scientific Sdn. Bhd) using primers T7F/ T7R (5’ TAATACGACTCACTATAGGG 3’/ 5’ GCTAGTTATTGCTCAGCGG 3’) of the selected colonies. Sequencing results were aligned and analyzed using using BioEdit software (Hall, T.A, 1999) to confirm insertion for subsequent *PfK13*-N537I and *PfK13*-V494I protein mutation.

### *In-vitro* expression and purification of recombinant *PfK13*-N537I and *PfK13*-V494I proteins

The confirmed plasmid DNA of *PfK13*-N537I and *PfK13*-V494I was transformed into *E*. *coli* Rosetta (DE3), and a single colony was chosen for the subsequent protein expression stage. The process starts off by introducing the colony into Lysogeny Broth (LB) supplemented with 100 μg/ml of Ampicillin and Chloramphenicol, followed by overnight incubation at 37°C. The following day, 1% of the overnight culture was inoculated into fresh Terrific Broth (TB) containing 100 μg/ml of ampicillin and chloramphenicol. The culture was incubated until the OD600 reached 0.6, at which point 1 mM of IPTG was added to induce protein expression. After the induction, the culture was continued incubated at 28°C for 18 hours. The cell supernatant was collected the next day with 30 minutes of centrifugation at 16,000 rpm at 4°C. Cell lysis was performed through the sonication method, comprising a 30-second cycle of sonication followed by a 30-second rest on ice, totaling 5 minutes. Bugbuster (Merck) was utilized as the buffer during this process.

The Immobilized Metal Affinity chromatography (IMAC) purification method was performed at the Malaysia Genome & Vaccine Institute (MGVI), National Institutes of Biotechnology Malaysia (NIBM). It is a common technique used to purify recombinant proteins fused to a short peptide affinity tag such as His-tagged. The expressed tagged-protein was purified using AKTA Avant 150 with HisTrap HP 1ml column. The binding buffer comprised 20 mM Tris, 150 mM NaCl, and 30 mM imidazole at pH 8.0. In addition, an elution solution containing comparable components but with 300 mM imidazole was used. Throughout the process, a constant flow rate of 1.0 mL/min was maintained. The elution columns with probable protein peaks were combined and concentrated using VivaSpin centrifugal concentrators and quantified using Nanodrop (Fisher Scientific). These approaches were required for the acquisition of pure his-tagged *PfK13*-N537I and *PfK13*-V494I protein mutants (His-tagged were obtained via the 2B-T cloning vector), which were then used in future investigations to study the binding relationship between protein mutants and ART.

### Purified protein analysis using SDS-PAGE gel and Orbitrap tandem mass spectrometry

Protein samples were separated using Sodium Dodecyl Sulphate- Polyacrylamide Gel Electrophoresis (SDS-PAGE) with Bolt 4–12% Bis-Tris Plus Pre-cast Gels (Thermo Fisher Scientific) and a novex™ Bolt Mini gel cassette (Life Technologies) in MOPS (Nu-PAGE™ 3-[N-morpholino]-propanesulphonic acid) running buffer at 100 Volts (V) using an EPS 500/400 DC Power Supply (Pharmacia). Forty μl (20μl) of 4X Laemmli sample buffer (100 mM Tris HCl [pH 6.8], 200 mM dithiothreitol [DTT], 4% sodium dodecyl sulphate [SDS], 20% glycerol, 0.2% bromophenol blue) were added to the samples and mixed, then the samples were heated at 95oC for 5 min. Precision Plus™ Protein standards (Biorad) were used as size markers. The separated proteins were visualized by staining with Coomassie blue (0.1% [w/v] Coomassie Brilliant Blue R-250, 45% [v/v] methanol, 10% [v/v] acetic acid) and immunoblot. Resolved SDS-PAGE gel was transferred onto nitrocellulose membrane for 1–2 hours at 30V or overnight at 10V at room temperature. The nitrocellulose membranes (Whatman Protran) were immersed in blocking buffer (1% bovine serum albumin [BSA] in PBST) for 30 to 60 min at room temperature or overnight at 4oC with constant slow agitation. The membrane was incubated in the primary antibody ἀ-*PfK13* (antibodies to peptides *PfK13* protein were raised in Rabbit by ABclonal Technology® (Woburn, USA)) for 1 h and washed with PBST 3 times at 10 min intervals. The membrane was incubated with an appropriate secondary antibody ἀ-rabbit IgG (BioRad; Lot no:147494) for 1 h and washed as before. To visualize protein bands, the membranes were immersed in enhanced chemiluminescence substrate solution (ECL, GE Healthcare) for 10 min and the protein band signal was detected by exposing the blot to GBox Chemi Documentation System (Syngene) for an appropriate amount of time before images being captured and analyzed. Additionally, to verify the results, the sample underwent analysis using the Thermo ScientificTM ExactiveTM Plus EMR OrbitrapTM LC-MS System at MGVI, NIBM, Malaysia. The protein sample was digested using the Trypsin-LysC enzyme (Mass Spec Grade, Promega) and 1.0 μl of concentrated trifluoroacetic acid (TFA, Sigma-Aldrich) was added to halt the reaction, followed by liquid chromatography analysis using The Dionex Ultimate 3000 RSLCnano (Thermofisher Scientific). The peptides were then identified using Orbitrap Fusion (Thermo Fisher Scientific).

### Binding interactions analysis using Isothermal Titration Calorimetry (ITC) and fluorescence spectroscopy

Each protein in the ITC method was subjected to two repetitions using 10 μM of protein. Simultaneously, ITC involved the utilization of 100 μM of Dihydroartemisinin (TargetMol) as an alternative to artemisinin (ART). Subsequently, the identical sample was employed for fluorescence spectrometry following the completion of the ITC procedure. The first step of this method was to exchange the buffer of the sample solution to 1% DMSO in phosphate buffer and dilute to different concentrations to optimize the procedure. A blank for this experiment was prepared using phosphate buffer with 1% DMSO. The *PfK13* protein and ligand, ART binding interaction data were collected and integrated using NanoAnalyze software, which come with the instrument. The binding of the ligand to the protein produced three parameters which are stoichiometry (n), association constant (K) and standard enthalpy change (Hb).

Finally, the formula of ΔG° = -RT ln K and TΔS° = ΔHb—ΔG° (if ΔHb = ΔH°) were used to calculate the changes in standard free energy, ΔG° and changes in entropy at a constant temperature, ΔS°, respectively. This approach entails analyzing the thermodynamic reaction in relation to binding energy, which reflects the affinities between *PfK13* protein mutants and ART. This aids in comprehending how point mutations affect the efficacy of the ART drug in treating malaria.

**Fig 1:**
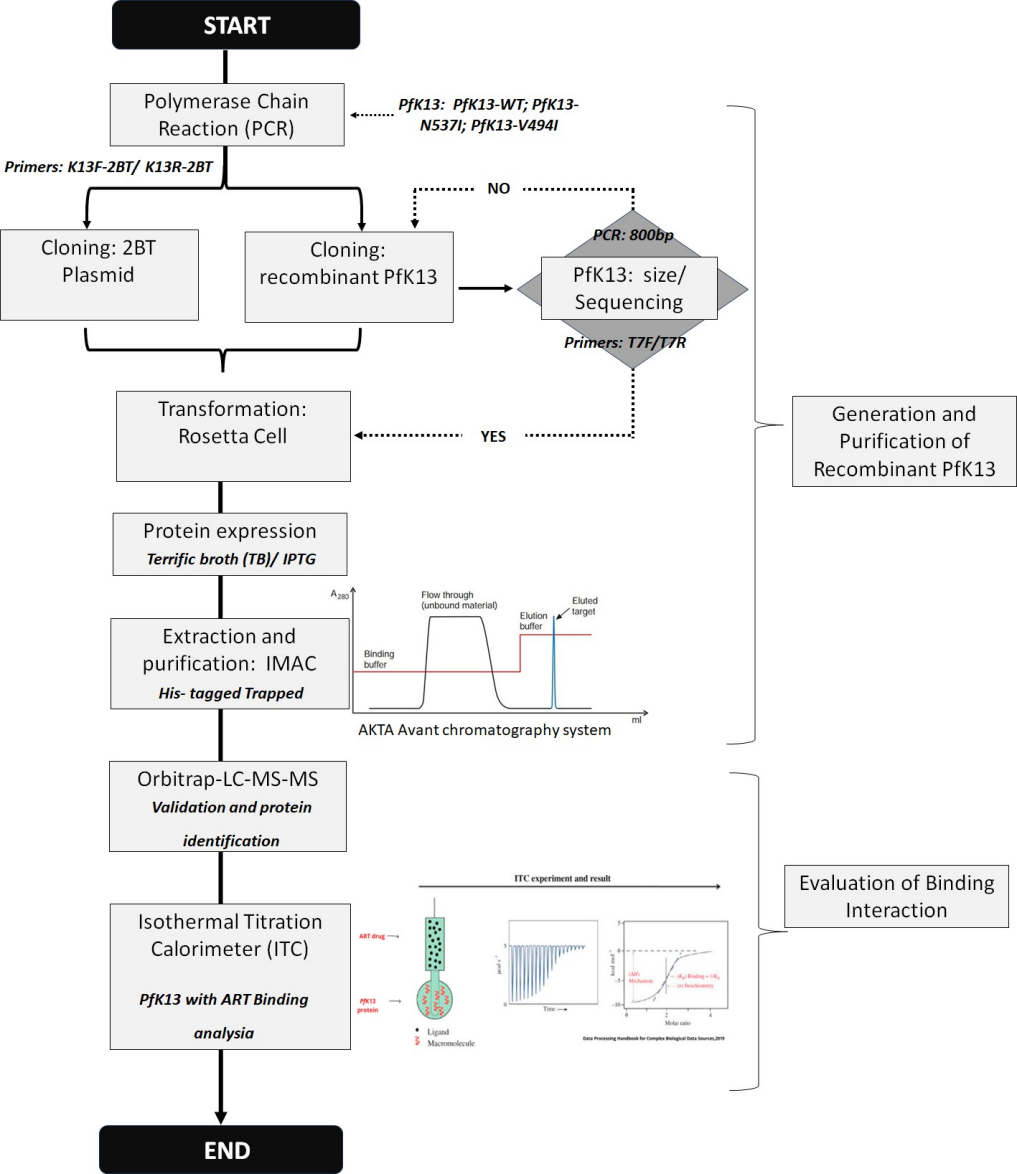
Illustrate the flowchart of methodology used in this study. The methodology was separated into 2 parts which were Part-1 was for the generation of *PfK13* recombinant protein and Part- 2 for analysis of binding between *PfK13* mutated protein and wild-type *PfK13* with ART respectively.

The results were further analyzed using fluorescence spectrometry. A Cary Eclipse Fluorescence Spectrophotometer (Agilent) was used in this method with the temperature maintained at 25°C. The function of this method is to analyse aromatic compound on the surface of protein such as tyrosine (Tyr), tryptophan (Trp) and phenylalanine (Phe). The procedure was conducted at MGVI, NIBM. The sample volume used was 300 μl with the wavelength range set 305–400 nm. Meanwhile, the excitation and emission slit were maintained at 5 nm and the voltage was set at 800 volts. A scanning speed of 100 nm/min was selected to expedite spectral acquisition. Smoothing was applied using a moving average with a factor of 9. The results obtained from this analysis aid in elucidating alterations in the binding interaction between *PfK13* protein mutants and ART, enhancing our comprehension of how point mutations in *PfK13* protein can induce ART-R and ultimately, clarifies the role of *K13* in the mechanism of ART-R. A summary of methodology was depicted in Fig 1.

## Results and discussion

### *PfK13*-N537I and *PfK13*-V494I protein expression and purification

**Fig 2:**
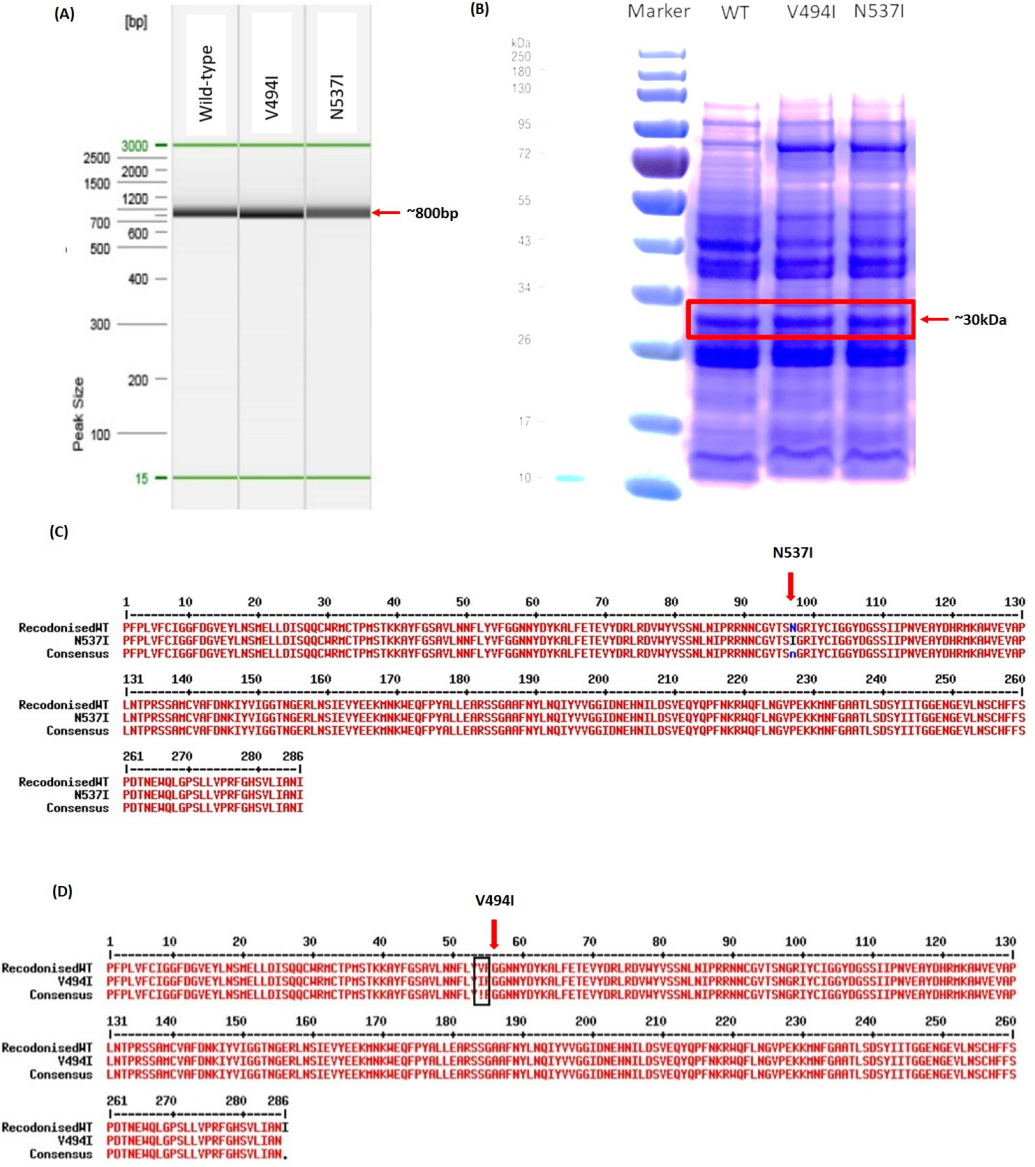
The fig illustrate *PfK13* protein expression. (A) The PCR amplification product visualized through Capillary Electrophoresis (Qiaxcel; Qiagen) displays a product size of approximately 800bp, corresponding to the *PfK13* propeller region. (B) SDS-PAGE reveals a band at around 30kDa (Red-box), corresponding to the *Pf K13*-WT, *PfK13*-N537I and *PfK13*-V494I propeller region protein respectively. Sequencing results for *PfK13*-N537I (C) and *PfK13*-V494I (D) were aligned using Multalin Software (Multiple sequence alignment by Florence Corpet (Multalin)). The sequences indicate the mutated regions for each respective protein expression.

Recombinant plasmids expressing *PfK13*-WT, *PfK13*-N537I, and *PfK13*-V494I proteins were successfully constructed using PCR and confirmed through SDS-PAGE and sequencing. The genes produce for *PfK13*-WT, *PfK13*-V494I, and *PfK13*-N537I were approximately 800bp, aligning with the size of the *PfK13* propeller region (Fig 2A). The *PfK13*-WT, *PfK13*-V494I, and *PfK13*-N537I protein was successfully expressed in the recombinant forms at a satisfactory level; their estimated sizes were 30 kDa (Fig 2B) and confirmed by sequencing (Fig 2C and 2D).

While we have achieved successful expression of *PfK13*, it remains challenging due to its notorious difficulty in expression. Scientists face hurdles when attempting to express *P*. *falciparum* proteins in heterologous systems, primarily due to the absence of an efficient recombinant protein synthesis system. This limitation not only affects the initial synthesis and characterization of proteins but also hinders functional studies and downstream assessments for purposes such as drug discovery. What is necessary are optimized protein expression systems capable of translating A/T-rich malaria parasite genes, often encoding low-complexity amino acid sequences, into well-folded proteins of high quality. These characteristics have been identified as significant factors impeding *P*. *falciparum* protein expression in heterologous cell-based systems [42, 43]. Host *E*. *coli* Rosetta (DE3) was selected as the production method for recombinant *PfK13* due to several advantages. These include *E*. *coli*’s ease of handling, safety, and genetic traits, which render it an appealing expression host [44]. Additionally, *E*. *coli* is well-known and will accept foreign DNA when subjected to genetic manipulation techniques. The advantage of being able to produce large amounts of protein efficiently and cheaply makes it more attractive than other options such as yeast or mammalian cell expression [45, 46].

During the expression process, optimal conditions for achieving the highest yield of recombinant *PfK13* included the use of terrific broth, incubation for 18 hours at 28°C, induction with 1.0 mM IPTG, and continuous shaking at 200 rpm. This outcome aligns with findings from previous studies [47, 48] indicating that variations in conditions such as temperature, IPTG concentration, or media composition did not significantly impact protein expression yield and solubility. Recombinant *P*. *falciparum* protein serves as an ideal alternative for the sustained production of substantial amounts of *PfK13*, given the challenges associated with long-term *P*. *falciparum* culture and the complexity of extracting and synthesizing parasite lysate protein.

### Protein purification and analysis

**Fig 3:**
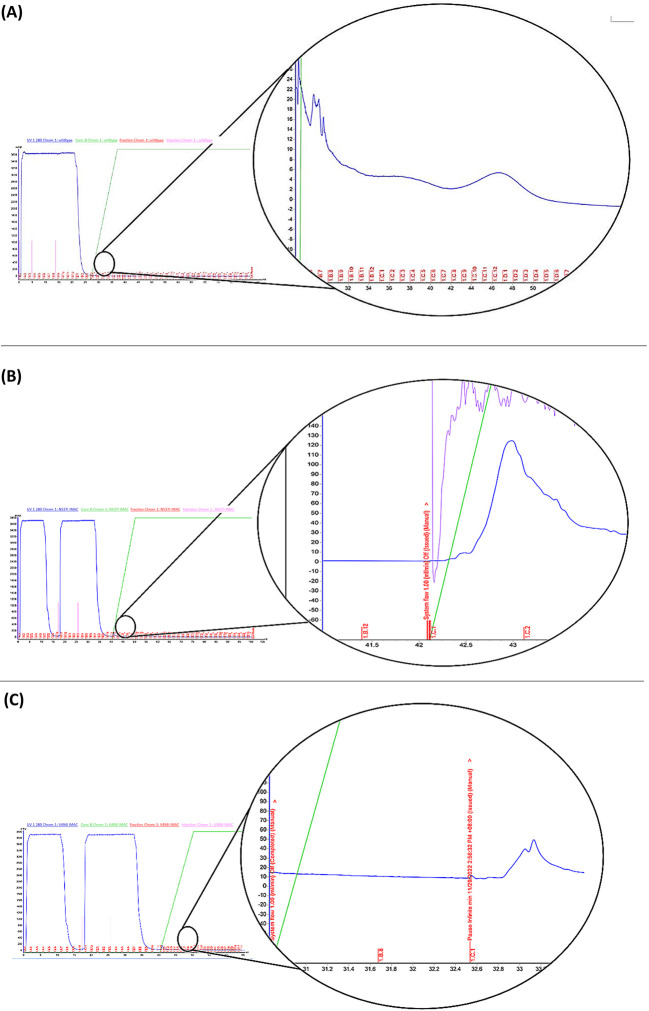
Protein purification by IMAC. Blue line indicates the quantity of His-tagged of each protein at the absorbance of 280nm wavelength. The x-axis illustrates the elution volume in milliliters, while the y-axis represents the absorbance measured in milli absorbance units (mAU). Full and zoomed version (black circle) of the chromatogram result of; (A) *PfK13*-WT sample, (B) *PfK13*-N537I and (C) *PfK13*-V494I. Fractions with visible peaks were collected and characterized using SDS-PAGE. Fraction with visible peaks was collected and characterized using SDS-PAGE (S1(A)–S1(F) Fig).

IMAC method was used to purify the *K13*-expressed protein. Result showed the recombinant *PfK13*-WT, *PfK13*-V494I and *PfK13*-N537I (Fig 3; S1(A)–S1(F) Fig) were able to successfully exhibit a sufficient level of expression of the *PfK13* propeller domain protein. The black circle shows the zoomed-in version of the chromatogram in which the peaks show the eluted protein that was bound to the His-tag. The elevated absorbance reading at the beginning, reaching 3000 mAU, posed a challenge in deducing the chromatography peaks’ full version due to the high protein content in *E*. *coli* Rosetta cells (DE3). All purified fraction were obtained and eluted from the peak as shown in the black circle. The sample’s absorbance was measured at 550 mAU, and the concentration of the pooled fractions, as determined by using nanodrop. *PfK13*-WT protein yield was 0.3 mg/ml, meanwhile for *PfK13*-N537I and *PfK13*-V494I, proteins was eluted at 0.28 mg/ml (125mAU) and 0.18 mg/ml respectively (45mAU) respectively. All eluted proteins exhibited a favorable 260/280 ratio, measuring less than 0.6, indicating minimal nucleic acid contamination.

**Fig 4:**
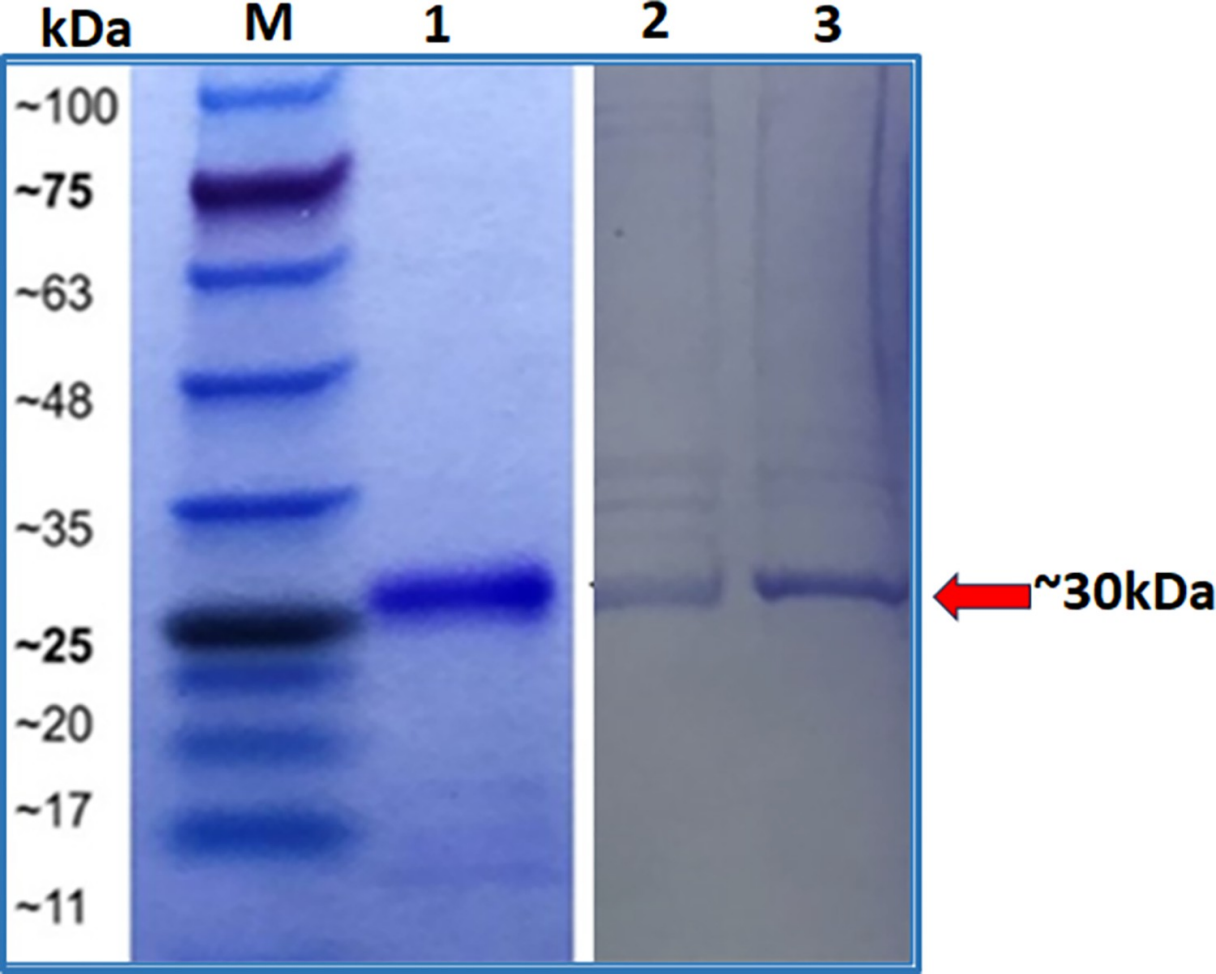
Purified protein. SDS-PAGE stained with Brilliant Coomasive Blue®. The *PfK13*s protein was purified using the IMAC system. Lane M- Prestained Protein Standard (Bioline) range (10-250kDa); Lane 1- *PfK13*-WT; Lane 2- *PfK13*-N537I and Lane 3- *PfK13*-V494I.

To authenticate the eluted proteins, SDS-PAGE gel analysis and Orbitrap tandem mass spectrometry were performed. The eluted fractions from the IMAC method have the band size of all *PfK13* propeller domain proteins with an approximate size of 30kDa (Fig 4). This suggests successful expression of both the mutants and wild-type recombinant *PfK13* proteins in the bacterial system. The accuracy of the observed band size was subsequently validated through Orbitrap tandem mass spectrometry at MGVI, NIBM. Identification of *PfK13*-WT protein using Orbitrap tandem mass spectrometry analysis showed a minimum false discovery rate (FDR) which means the q-value of zero indicates that at this score, the probability of being incorrect is 0%. (Table 1). Peptides indicate the number of peptides match found in the database. Meanwhile, the unique peptides column means a peptide regardless of length is found only in one protein in a proteome of interest, even though it may present more than once in that protein [49]. Similar results were observed among the *PfK13*-N537I and *PfK13*-V474I (Table 1).

**Table 1:** Analysis of *PfK13*-WT, *PfK13*-N537I and *PfK13*-V494I with Orbitrap tandem mass spectrometry analysis.

No.	Description	Exp. q-value	Peptides	Peptide Sequence Matches	Unique peptides
*PfK13*-WT	Kelch protein 13 (Fragment) OS^1^ = *Plasmoduim falciparum* OX^1^ = 5833 GN^1^ = *K13* PE^1^ = 4 SV^1^ = 1	0	10	80	1
*PfK13*-N537I	Kelch propeller domain (Fragment) OS^1^ = *Plasmodium falciparum* OX^1^ = 5833 GN^1^ = *K13* PE^1^ = 4 SV^1^ = 1	0	7	34	2
*PfK13*-V474I	Kelch protein OS = *Plasmodium falciparum* OX = 5833 PE = 4 SV = 1	0	1	8	1

^1^OS = Oligosaccharides, OX = organism identifier, GN = gene name, PE = protein

*PfK13*-WT protein has zero experimental q-value. There were 10 peptides and the identified proteins obtained 1 unique peptide. Meanwhile, *PfK13*-N537I for Orbitrap tandem mass spectrometry analysis. *PfK13*-N537I exhibited a q-value of zero in the experiment, with 7 peptides found, including 2 unique peptides. Subsequently, for *PfK13*-V494I using Orbitrap mass spectrometry, a single *K13* protein fragment with a q-value of zero was identified, and only one peptide, along with one unique peptide, was detected in this sample. The obtained results are in line with expectations, validating the presence of Kelch 13 protein in the purified sample obtained through IMAC. This corresponds with a prior study that utilized mass spectrometry to verify the presence of *PfK13* protein in their samples following the purification step [50]. The purified protein, once confirmed, proceeded to the binding analysis utilizing both ITC and fluorescence spectroscopy. The subsequent phase of this study involved analyzing the binding interaction between *PfK13* protein mutants and ART using ITC and fluorescence spectroscopy to evaluate the impact of mutations on the efficacy of ART drug.

### Evaluation of binding interactions between *PfK13* recombinant proteins and ART drug

**Fig 5:**
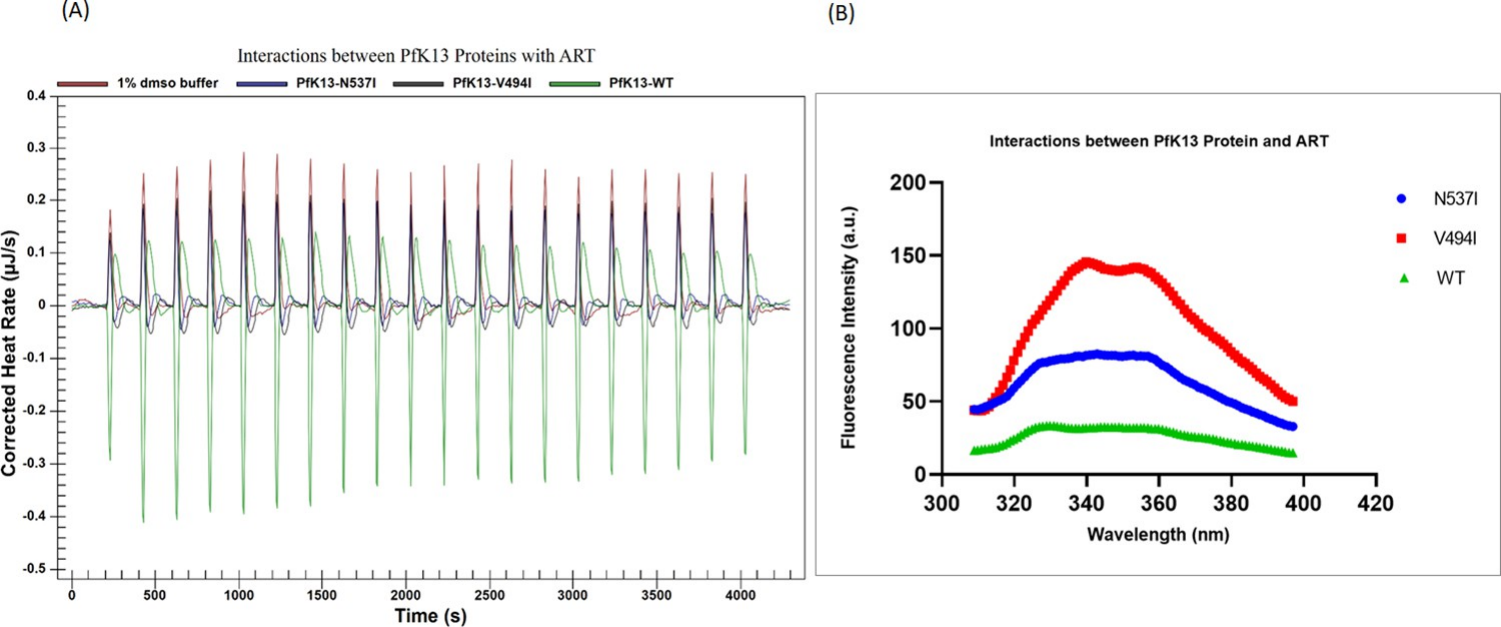
Binding evaluation. (A) ITC after interacting with ART in 1% dimethyl sulfoxide (DMSO) buffer (red), *PfK13*-N537I (blue), *PfK13*-V494I (black) and *PfK13*-WT (green); (B) Fluorescence intensity of *PfK13* proteins post-binding with ART using ITC (*PfK13*-N537I-blue), *PfK13*-V494I (red) and *PfK13*-WT (green).

The ITC method was employed to investigate binding interactions between proteins and ligands by measuring the endothermic or exothermic reactions generated during the binding of these two molecules. This approach enables the calculation of the binding constant, providing insights into binding affinities and contributing to the assessment of the efficacy of ART in malaria treatment [51]. From the graph produced by this method, changes in enthalpy and binding constant can be calculated [52]. Fig 5, shows the combined results of binding interaction *PfK13*-WT, *PfK13*-V494I and *PfK13*-N537I.

The trend of *PfK13*-WT during the binding interaction was an endothermic reaction compared to the other two proteins, *PfK13*-V494I and *PfK13*-N537I which shows an exothermic reaction trend (Fig 5A; S1 Table). However, enthalpy changes of this protein cannot be deduced since the sigmoidal curve cannot be constructed. The generation of an optimized sigmoidal curve proved challenging due to the use of a DMSO solution to dissolve the ART drug resulted in heat spikes during the initial stages, hindering the achievement of optimized heat measurements and preventing the reaction from reaching saturation. Moreover, a 100x dilution was necessary to reduce the concentration of the ART drug in DMSO to 1%, and a similar process was required for the purified protein. Unfortunately, this process led to a significant loss of purified protein, preventing the production of a sigmoidal curve and, consequently, the deduction of the binding constant.

Technically, the ITC instrument measured any heat produced during the interaction of these two molecules [53]. This method is very sensitive to substances that can produce any heat [54]. DMSO is one of the substances that can easily produce heat and most ART drug available in the market has the highest solubility in DMSO, causing a technical problem during the procedure [55]. This condition is unfavorable for ITC since DMSO dilution heats are extremely high and may obscure reaction heats [54]. From the result of the previous study [47], the hexameric assemblies of *PfK13*-WT and other mutants did not generate any heating and significant changes in the solution shape hence, proving that the result of ITC for the WT sample is endothermic. bioreactor could prove beneficial in optimizing the ITC method, allowing for the dilution of DMSO concentration as a buffer in the process. Nevertheless, various engineering parameters, including vessel design, impeller geometry, tip speed, mixing time, oxygen transfer rate, volumetric mass transfer coefficient, and power number, need consideration. Nevertheless, to validate the results obtained through the ITC method, fluorescence spectroscopy was utilized to gain a more profound understanding of how point mutations on the *PfK13* protein affect the binding interaction with ART. This investigation seeks to clarify potential compromises in the effectiveness of ART in treating malaria, potentially resulting in the development of ART-R.

The highest fluorescence intensity was *PfK13*-V494I followed by *PfK13*-N537I and *PfK13*-WT (Fig 5B; S2 Table). The intensity of aromatic compounds like tyrosine and tryptophan was assessed using this approach. When the *PfK13* protein binds with ART, the reduction in protein surface exposure results in fewer aromatic compounds being detected. This will serve as potential evident that the fluorescence intensity of both protein mutants, *PfK13*-V494I and *PfK13*-N537I, exceeded that of *PfK13*-WT. This indicates high affinity between ART and *PfK13*-WT, while the two mutations exhibit lower affinity, implying increased exposure to aromatic compounds.

The finding of this study supports the proposed mechanism underlying the use of artemisinin (ART) to treat malaria, which holds that ART increases parasite oxidative stress and causes protein degradation through the unfolded protein response process [40]. Additionally, this work supports the finding of another study that ART functions as a dual-action agent on proteins, causing damage and unfolding while blocking proteasome activity, which accumulates ubiquitinated proteins [56]. However, this route is interfered with by a particular mutation in the *PfK13* protein, which modifies the protein’s structure and interferes with its ability to connect with ART. The emergence of ART resistance is caused by this change, which enables the parasite to withstand ART treatment.

In essence, this study aimed to elucidate the mechanism behind the potential origin of artemisinin resistance. Recombinant protein mutants and WT of *PfK13* were created with the primary aim of producing sufficient soluble proteins for subsequent in vitro studies on protein-drug interactions. The expressed proteins underwent purification and were further examined for their interactions with the ART drug through methods including ITC and fluorescence spectroscopy. The introduction of point mutations into the *PfK13* protein has affected its interaction with ART, resulting in structural changes that could impact binding affinity. These alterations were identified through a range of biophysical analyses, including ITC and fluorescence spectroscopy. In the context of thermodynamic analysis, the binding interaction between *PfK13* protein mutants and ART exhibits an exothermic reaction, releasing heat during the interaction, which differs from *PfK13*-WT. *PfK13*-WT, in contrast, demonstrates an endothermic reaction, absorbing heat. This indicates that a point mutation in the *PfK13* protein induces alterations in the binding interaction, potentially influencing the effectiveness of ART in malaria treatment. Fluorescence spectroscopy, used to assess aromatic compounds on the protein surface, was employed to corroborate the findings from ITC. High affinity binding between the protein and ART results in a reduction of exposed protein, consequently decreasing fluorescence intensity. The findings revealed an increased fluorescence intensity in *PfK13* protein mutants when contrasted with *PfK13*-WT, implying that the point mutation might hinder the optimal binding of ART, leading to a reduction in affinity binding. This potential disruption could compromise the efficacy of ART in malaria treatment by interfering with its function in the ubiquitination pathway. As a result, the parasite might evade damage during ART treatment, ultimately leading to ART-R. This research study could serve as the initial step towards unraveling the function of *PfK13*, providing insights into the workings of the ART-R mechanism. This understanding can contribute to preventing the emergence of ART-R cases not only in Malaysia but also in other countries.

Considering the study’s findings and others, it is essential for the government to remain vigilant regarding malaria drug resistance issues. Urgent actions, particularly those undertaken by policymakers, are necessary to prevent the onset of ART-R cases in Malaysia. Preventive measures should involve monitoring high-risk malaria outbreak areas, particularly rural regions with limited access to malaria treatment through monitoring drug efficacy and surveillance to identify genetic marker associated with resistance, improvement of diagnostic tool for early diagnosis efficiency as well as strengthening health system, utilization of other drug combination for ACTs, continuous funding for research and development, implementation of vector control measures, monitoring cross-border movement and advance community engagement for awareness of malaria disease. A comprehensive approach is required to strategically prevent and control the emergence of ART-R, not only in Malaysia but also in other affected regions.

## Conclusions

This finding indicates that mutations in the *PfK13* protein can alter its binding interactions, resulting in structural changes that decrease its affinity for artemisinin (ART). This suggests that ART may be less effective in treating malaria, potentially contributing to the emergence of artemisinin resistance (ART-R). The study underscores the importance of comprehending the binding interactions between *PfK13* mutant proteins and artemisinin to address ART resistance in malaria. However, solely understanding these interactions is insufficient to prevent the development of ART-R. By utilizing a comprehensive strategy that integrates surveillance, prevention, treatment, research, and cooperation, we can address the challenge of artemisinin-resistant malaria and ensure the continued efficacy of artemisinin-based treatments in the long run. A comprehensive approach is required to strategically prevent and control the emergence of ART-R, not only in Malaysia but also in other affected regions.

## Supporting information

S1 Fig Protein purification by IMAC. (A) Wild type (WT) full image; (B) WT zoom in image; (C) *PfK13-N537I* full image; (D) *PfK13-N537I* zoom in image; (E) *PfK13- V494I* full image; (F) *PfK13-V494I* zoom in image.
(ZIP)

S1 Table Raw data of binding evaluation with ITC method. (A) Wild-type; (B) *PfK13-N537I*; (C) *PfK13-V494I*.
(XLSX)

S2 Table Raw data for fluorescence spectrometry showing fluorescence intensity of PfK13 proteins post-binding with ART. (A) PfK13-N537I; (B) WT; (C) PfK13-V494I.
(XLSX)
