# Supplementary figures and images for: Evaluation of the binding interactions between Plasmodium falciparum Kelch-13 mutant recombinant proteins with artemisinin
Source: PLoS One. 2024 Aug 15;19(8):e0306975. doi: 10.1371/journal.pone.0306975 (PMC11326563; doi:10.1371/journal.pone.0306975)

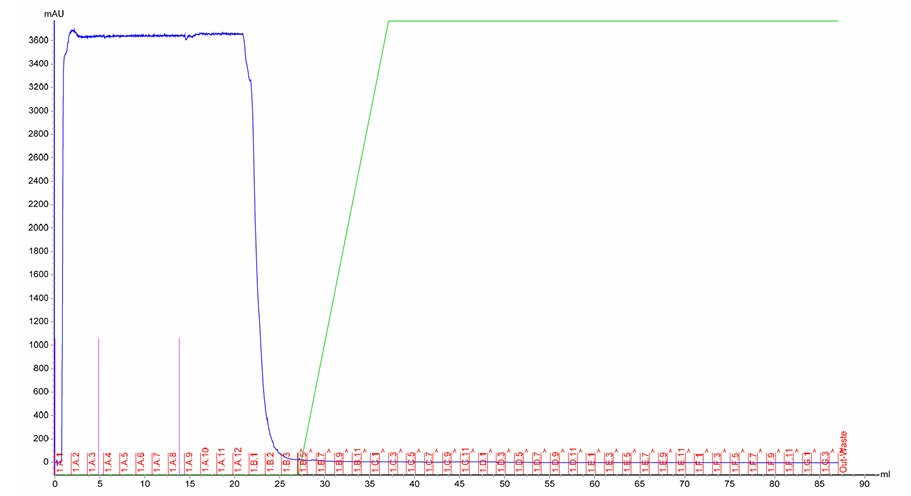

Supplement: S1 Fig — (A) Wild type (WT) full image; (B) WT zoom in image; (C) PfK13-N537I full image; (D) PfK13-N537I zoom in image; (E) PfK13- V494I full image; (F) PfK13-V494I zoom in image. (ZIP) [file pone.0306975.s001.zip › S1_Fig/S1 Fig (A)- WT full version.tif]

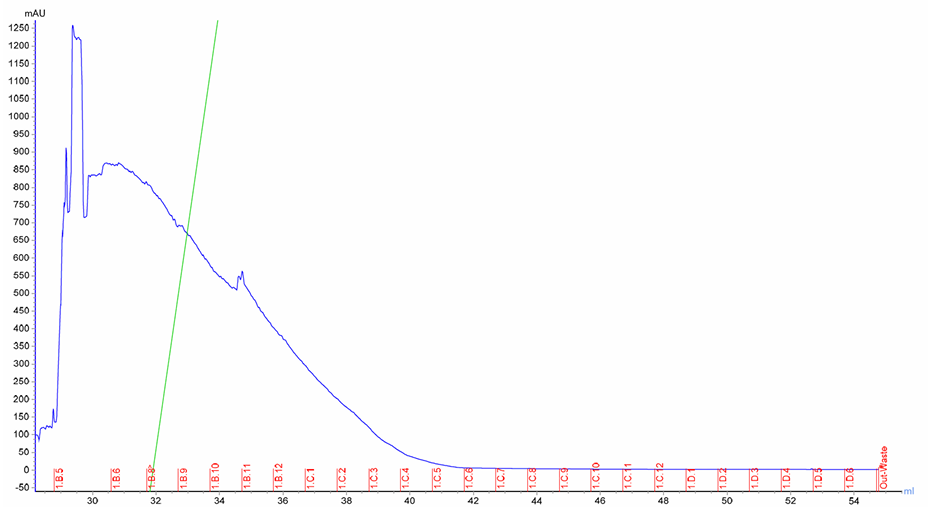

Supplement: S1 Fig — (A) Wild type (WT) full image; (B) WT zoom in image; (C) PfK13-N537I full image; (D) PfK13-N537I zoom in image; (E) PfK13- V494I full image; (F) PfK13-V494I zoom in image. (ZIP) [file pone.0306975.s001.zip › S1_Fig/S1 Fig (B)- WT zoom version.tif]

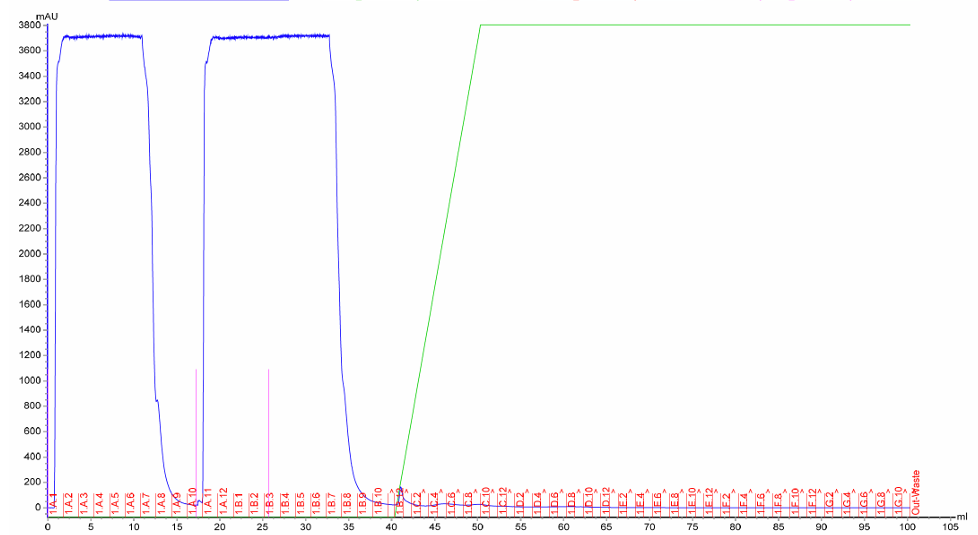

Supplement: S1 Fig — (A) Wild type (WT) full image; (B) WT zoom in image; (C) PfK13-N537I full image; (D) PfK13-N537I zoom in image; (E) PfK13- V494I full image; (F) PfK13-V494I zoom in image. (ZIP) [file pone.0306975.s001.zip › S1_Fig/S1 Fig (C)- N537I full version.tif]

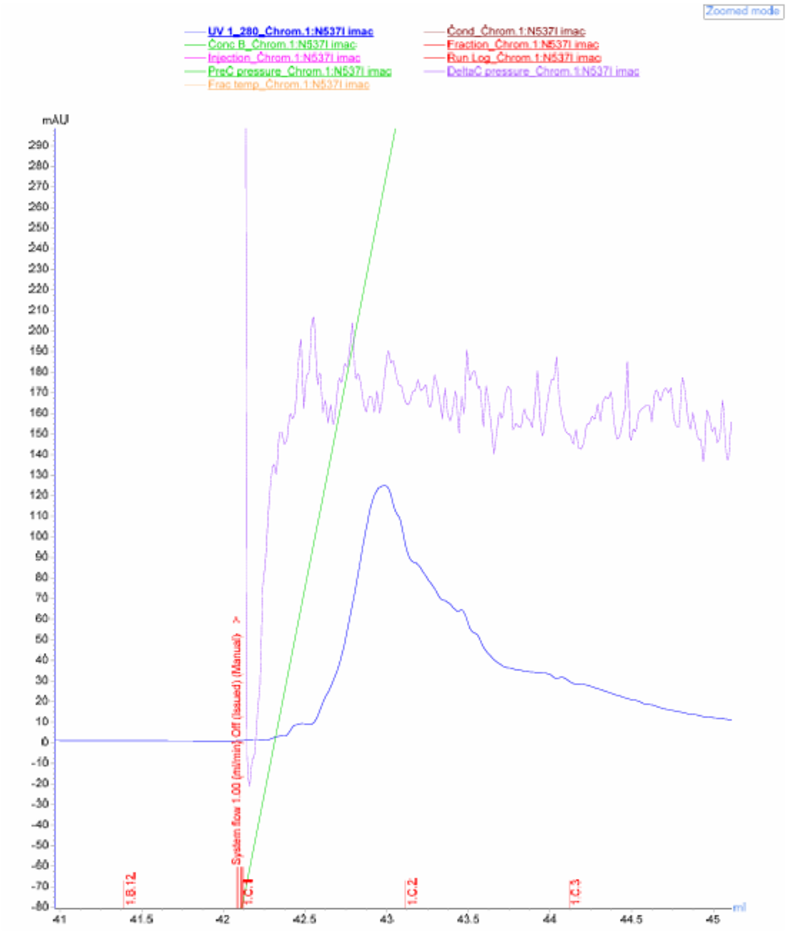

Supplement: S1 Fig — (A) Wild type (WT) full image; (B) WT zoom in image; (C) PfK13-N537I full image; (D) PfK13-N537I zoom in image; (E) PfK13- V494I full image; (F) PfK13-V494I zoom in image. (ZIP) [file pone.0306975.s001.zip › S1_Fig/S1 Fig (D)- N537I zoom version.tif]

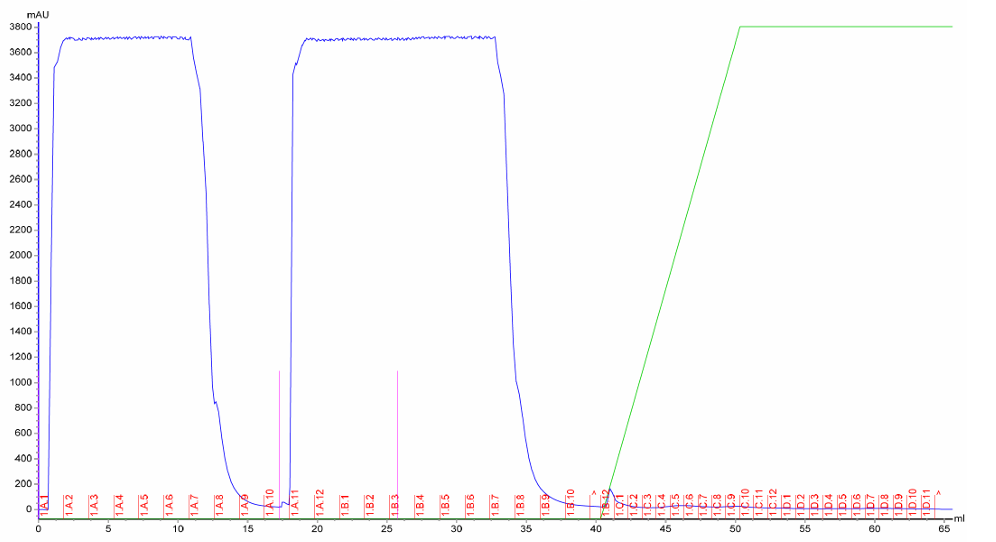

Supplement: S1 Fig — (A) Wild type (WT) full image; (B) WT zoom in image; (C) PfK13-N537I full image; (D) PfK13-N537I zoom in image; (E) PfK13- V494I full image; (F) PfK13-V494I zoom in image. (ZIP) [file pone.0306975.s001.zip › S1_Fig/S1 Fig (E)- V494I full version.tif]

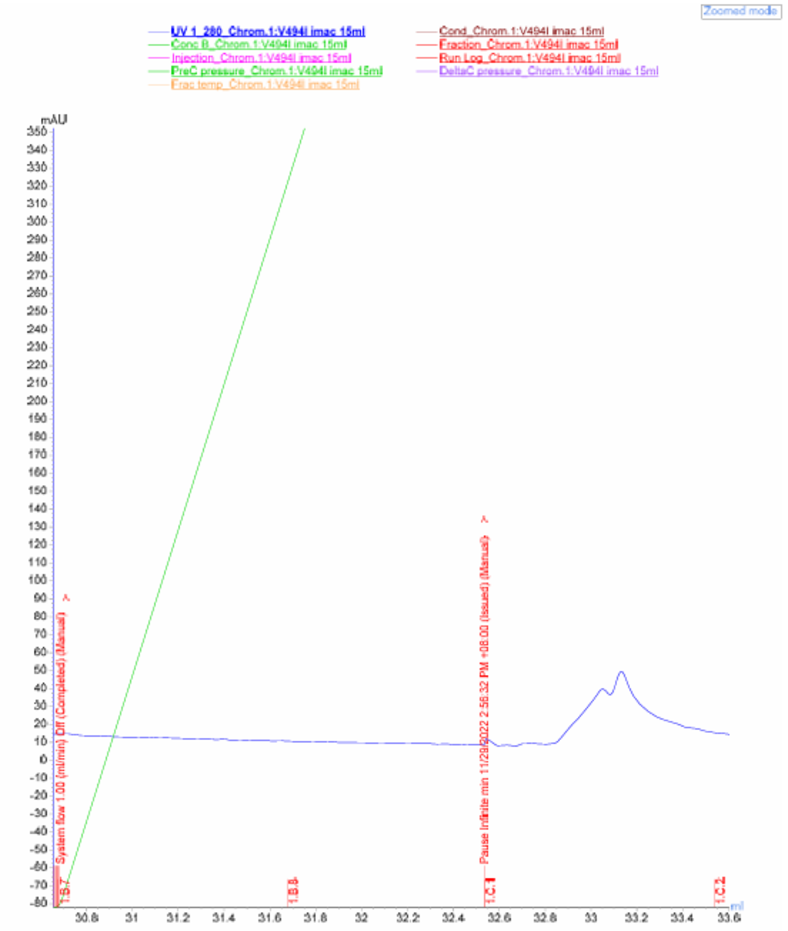

Supplement: S1 Fig — (A) Wild type (WT) full image; (B) WT zoom in image; (C) PfK13-N537I full image; (D) PfK13-N537I zoom in image; (E) PfK13- V494I full image; (F) PfK13-V494I zoom in image. (ZIP) [file pone.0306975.s001.zip › S1_Fig/S1 Fig (F) - V494I zoom version.tif]
